# Influence of perinatal distress on adverse birth outcomes: A prospective study in the Tigray region, northern Ethiopia

**DOI:** 10.1371/journal.pone.0287686

**Published:** 2023-07-13

**Authors:** Kebede Haile Misgina, Lindsay Levine, H. Marike Boezen, Afework Mulugeta Bezabih, Eline M. van der Beek, Henk Groen

**Affiliations:** 1 Department of Public Health, University of Aksum, Axum, Ethiopia; 2 Department of Epidemiology, Faculty of Medical Sciences, University of Groningen, Groningen, The Netherlands; 3 Perelman School of Medicine, University of Pennsylvania, Philadelphia, Pennsylvania, United States of America; 4 Department of Nutrition, College of Health Sciences, University of Mekelle, Mekelle, Ethiopia; 5 Department of Paediatrics, Faculty of Medical Sciences, University of Groningen, Groningen, The Netherlands; Arba Minch University, ETHIOPIA

## Abstract

**Background:**

In low-income countries, where socioeconomic adversities and perinatal distress are common, adverse birth outcomes are significant public health problems. In these settings, perinatal distress, i.e., high symptoms of anxiety, depression, and/or stress during pregnancy, may be linked with adverse birth outcomes. However, few prospective studies have investigated the impact of perinatal distress on adverse birth outcomes such as preterm birth (gestational age <37 weeks), low birth weight (<2.5 kg), and small for gestational age birth (birth weight below the 10^th^ percentile for gestational age and sex).

**Objectives:**

Our main objective was to assess the influence of perinatal distress on adverse birth outcomes. Secondly, to investigate if perinatal distress is an independent risk factor or a mediator in the pathway between socioeconomic adversity and adverse birth outcomes.

**Methods:**

In a prospective cohort study following 991 women from before 20 weeks of gestation until delivery in northern Ethiopia, we collected self-reported data on distress at a mean of 14.8 (standard deviation [SD] = 1.9) and 33.9 (SD = 1.1) weeks of gestation. Distress was measured using the Edinburgh Postnatal Depression Scale, the anxiety subscale of the Hospital Anxiety and Depression Scale, and the Perceived Stress Scale. To determine birth outcomes, gestational age was estimated from the last menstrual period, fundal palpation, and/or ultrasound, while birth weight was obtained from delivery records and measured within three days after birth for those delivered at home. Logistic regression and mediation analysis were employed to evaluate the impact of perinatal distress on adverse birth outcomes.

**Results:**

Perinatal anxiety (OR [95% CI] 1.08 [1.02, 1.13]), depression (1.07 [1.03, 1.11]), stress (1.14 [1.07, 1.22]), and total distress (1.15 [1.07, 1.23]) were all associated with low birth weight, and small for gestational age birth but none did with preterm birth. Mediation analysis demonstrated that perinatal distress was a mediator in the pathway between socioeconomic adversity and adverse birth outcomes.

**Conclusion:**

Our study revealed that perinatal distress was linked with adverse birth outcomes and acted as a mediator between socioeconomic adversity and these outcomes. Our findings highlight the importance of screening women for distress and providing appropriate interventions, focusing on women experiencing socioeconomic adversity. Integrating mental health services into primary maternal care in low-income countries could be an effective approach to achieve this.

## Introduction

In developing countries, adverse birth outcomes, which are defined as preterm birth (PTB, delivery before 37 completed weeks of gestation), low birth weight (LBW, weight below 2,500 g at birth), and/or small for gestational age (SGA, birth weight below the 10^th^ percentile for gestational age and sex), impose a heavy burden [1–3]. In 2014, approximately 14.8 million babies were born preterm, and in 2015, over 20.0 million had low birth weight and/or were small for gestational age [2–4]. The prevalence of adverse birth outcomes is highest in South Asia and sub-Saharan Africa [4]. Neonatal mortality, which accounts for 47% of deaths among children under five years of age, is primarily caused by adverse birth outcomes [5, 6]. Preterm birth is responsible for 35% of neonatal deaths, while low birth weight accounts for nearly 22% [4, 6].

Perinatal distress refers to high symptoms of anxiety, depression, and/or stress during the perinatal period, i.e., the period between 22 weeks of gestation and the end of the first week post-partum. In low-income countries, about 25% of women are affected by perinatal distress [7–10]. Perinatal distress may predispose women to inadequate prenatal care and low gestational weight gain [11]. It may also be linked with adverse birth outcomes such as preterm birth (defined as birth before 37 weeks of gestation), low birth weight (weight <2.5 kg), and small for gestational age birth (birth weight <10^th^ percentile for gestational age and sex) [7, 12, 13]. After birth, perinatal distress may affect mother-to-child bonding, [14, 15] exclusive breastfeeding [16], early childhood development [17–23], and health later in life and the health of future generations [19–21]. However, the association of perinatal distress with adverse birth outcomes has been reported inconsistently in reviews and meta-analyses, which calls for further investigation, especially in low-income countries [7, 24, 25].

The three domains of perinatal distress—perinatal anxiety, depression, and stress—are usually comorbid, and their co-occurrence has been posited to have a compounded influence on birth outcomes. Even so, the majority of previous studies in low-income countries have focused on a single aspect of distress, specifically depression, and have not considered anxiety and stress. Additionally, only a few studies have addressed the level of distress in any of the three domains through pregnancy and/or its potential impact on birth outcomes [26, 27]. For example, an increase in the level of distress over the course of pregnancy has been shown to affect birth outcomes. Regardless of the change over time, persistent high symptoms of distress may also impact outcomes [28]. Yet, studies prospectively assessing the influence of distress on birth outcomes in low-income countries are rare.

Perinatal distress and adverse birth outcomes also have several common risk factors, including poor economic status, food insecurity, intimate partner violence, and lack of social support [8, 29–35]. These shared risk factors raise the question if perinatal distress increases the risk of adverse birth outcomes independently or whether it is a mediator in the pathway of socioeconomic adversity to adverse birth outcomes. Socioeconomic adversity is defined as poor economic status, food insecurity, women’s disempowerment, intimate partner violence, lack of social support, and stressful life events. Therefore, assessing the causal mechanisms that underpin the association between distress and adverse birth outcomes is needed [24, 36] to design adequate interventions and improve perinatal health and birth outcomes in low-income countries.

Furthermore, most analyses have not controlled for important biomedical variables such as maternal nutritional status or socioeconomic adversity such as intimate partner violence and women’s disempowerment. The inconsistencies in the association between perinatal distress and adverse birth outcomes of the prior studies may be attributed to residual confounding [7, 24, 37]. Additionally, some previous studies have used a more general Self-Reported Questionnaire (SRQ-20) as a screening tool for perinatal distress, which included somatic symptoms that are also common during pregnancy. Thus, poor sensitivity of the screening tool in pregnant women might have affected the findings [29, 38]. In light of these limitations, inconsistencies, and knowledge gaps, there is a need for evidence that guides interventions to improve both the perinatal health and birth outcomes. Thus, the present study aimed to assess the influence of perinatal distress on adverse birth outcomes and examine if perinatal distress is an independent risk factor or a mediator in the pathway between psychosocial adversity and adverse birth outcomes.

## Methods

### Study design, setting, and population

The **KI**lite-Awlaelo **Tigray E**thiopia **(KITE)** cohort is a population-based prospective cohort study conducted in Kilite-Awilaelo Health and Demographic Surveillance Site (KA-HDSS) in the Tigray region of northern Ethiopia between February 2018 and January 30, 2019 [39]. The site consists of three urban and ten rural kebeles (the smallest administrative units) with approximately 110,000 inhabitants. Women of reproductive age account for 24% of the population, and 4,500 pregnancies per year are expected within the site. Most of the inhabitants live under rural conditions, and agriculture is the primary source of income. Ethiopia has a three-tier health care system with health posts at the forefront of primary care. Each kebele has one health post staffed by two to three health extension workers. Health posts provide promotional and preventive services under the umbrella of the ‘health extension package’ mainly at a household level. The package consists of 16 components including maternal health, family planning, nutrition, and sanitation [40].

### Sample size and sampling technique

The sample size for the KITE cohort was determined primarily based on the relationship between maternal nutritional status and birth outcomes. We used an estimated proportion of 24.6% low birth weight among women with mid-upper arm circumference (MUAC) ≥23 cm, and 32.6% among women with MUAC <23 cm [33]. With an alpha of 5% (2-sided), 80% power, and a 10% drop-out rate, the total sample size was calculated at 1,100. With this sample size, differences of more than 10% could be detected across wide-ranging prevalences and with varying ratios of exposed versus non-exposed. For continuous outcomes, effect sizes >0.2 standard deviations could be detected.

From the non-pregnant women (n = 17,500) living in the study area whose weight was measured between August and October 2017, identification of pregnant women took place by applying different methods [39]. The methods include a community-based survey by Health Extension Workers through the “Women Development Army”, a network of health information workers reaching individual households around the health posts. The records of the nearby antenatal clinics and the KA-HDSS database were also used. All eligible pregnant women identified between February and September 2018 were included consecutively and followed until delivery. Married women, aged 18 or older, and who completed ≤20 weeks of gestation, were eligible for the study.

### Data collection tool and procedure

Data were collected by qualified health extension workers through oral interview and anthropometric measurements, supplemented with data extracted from the KA-HDSS database and antenatal records. The questionnaire was adapted from the literature [33, 41–43] and pre-tested on 55 women in Tahtay-Maichew, Tigray region. Details on the measurement of the data collected at different time points are provided below.

### Measurement

#### Maternal socioeconomic, reproductive, and dietary characteristics

Age in years, residence, religion, educational status, occupation, parity, household size, and economic status were extracted from the KA-DHSS database. The surveillance site updates the database every six months except for wealth index. Adjustments were made at inclusion when there is a change in wealth index since the last update. Economic status was assessed by asking about housing characteristics, access to improved sources of drinking water and sanitation facilities, and ownership of household assets, land, and livestock. Subsequently, principal component analysis was used to generate wealth index quintiles designating the lowest to the highest economic status [44].

Data on health extension package implementation were collected at inclusion by determining whether the women’s households were certified as model households. A model household was defined as a household that received short-term training and implemented the package after the training [40]. Furthermore, self-reported history of pre-pregnancy illnesses, including chronic non-communicable diseases, and work burden rated as easy, moderate, or difficult were collected at inclusion.

Partner support was measured using the five-item Turner Support Scale, each rated from 0 to 3 [45], with scores of <10 indicating low support. Similarly, support from other social sources was obtained using Oslo-3 Social Support Scale at inclusion [46], with total scores in the range of 3 to 14, and scores ≤8 being defined as low [46]. Totaling the two measures of support at inclusion, a total social support score was created, and low total social support was defined as low support from partner and other social sources.

As for the reproductive characteristics assessed, parity, history of abortion, and history of stillbirth were extracted from the KA-DHSS database. Also, history of preterm birth, delivery by Caesarean section, and severe perinatal hemorrhage were collected by interview at inclusion. Based on this information, a history of adverse pregnancy outcomes was defined as having experienced one or more of the following: abortion, stillbirth, preterm birth, severe perinatal hemorrhage, or delivery by Caesarean section. Additionally, women were asked at inclusion if they wanted to get pregnant at the time they became pregnant, wanted later, or did not want at all. Accordingly, index pregnancy that was wanted later or not wanted at all was considered unplanned.

Moreover, women were asked the four-item Hurt, Insult, Threaten, and Scream (HITS) questions, each rated on a scale from 1 to 5 to measure intimate partner violence at inclusion, with a total score of ≥11 indicating violence [47]. To assess women’s empowerment, participants were asked nine questions addressing five domains at inclusion: 1. earning and control over income (relative income to husband, control over men’s income, and control over women’s income); 2. decision-making on household purchases; 3. mobility and health care autonomy (decision-making on family visits, and women’s health); 4. attitude towards domestic violence; and 5. ownership of assets (farmland and house) [41, 48]. By coding each positive response as 1 and adding the responses, a women empowerment score ranging from 0 to 9 was obtained. By assigning each domain an equal weight (1 point each) to be shared by the indicators within the respective domains, women who scored ≥80% or at least 4 out of 5 were considered empowered [49].

Food and dietary characteristics, including frequencies of alcohol and coffee intake, fasting, dietary diversity and food security were assessed at inclusion. According to the 2016 FAO guideline, dietary diversity was assessed by asking women about consuming a list of foods over a 24 hours period with ‘yes’ or ‘no’ as the answer options. The list was organized into ten groups: grains, white roots, and tubers; pulses; nuts and seeds; dairy; meat, fish and poultry; egg; dark green leafy vegetables; other vitamins A-rich fruit and vegetables; other fruit; and other vegetables. Scoring five or more groups was defined as adequate diet diversity [42].

To assess food security using the Household Food Insecurity Access Scale, women were queried how often nine specific food insecurity-associated conditions, if any, happened in the past month, categorized as 0) never, 1) rarely, 2) sometimes, or 3) often [43]. Their sum yielded a food insecurity score ranging from 0 to 27. A household was classified as food secure if the response to all occurrence questions was ‘no’ or if the only ‘yes’ response concerned the question “did you worry that your household would not have enough food” and the frequency of occurrence was ‘rarely’. All other households were classified as food insecure [43].

#### Maternal anthropometric characteristics and nutritional status

Maternal weight, height and blood pressure were measured in duplicate at inclusion and 32 to 36 weeks of gestation as per standard techniques, using a weight scale (to the nearest 100 g), height-measuring board (to the nearest 0.1 cm), and a mercury sphygmomanometer (to the nearest 0.5 mmHg). Of note, height was measured at inclusion only. Pre-pregnancy BMI (pre-pregnancy weight [kg])/[height (m)]^2^) was categorized as underweight (BMI <18.5), normal (BMI = 18.5 to 24.9) or overweight (BMI ≥25.0). Hypertension was defined as blood pressure ≥140/90 mmHg. Also, adequacy of gestational weight gain (weight at 32 to 36 weeks–pre-pregnancy weight) was classified as per the 2009 Institute of Medicine guideline [50].

#### Obstetric characteristics during the index pregnancy

Self-reported stressful life events that occurred over the past year, illness during pregnancy, pregnancy complications, prenatal care, and delivery details were obtained at 32 to 36 weeks and at or immediately after birth, as appropriate [51]. For women who began prenatal care ≤16 weeks of gestation, prenatal care was defined as adequate plus (five or more visits), adequate (four visits), or intermediate (two to three visits). Prenatal care was considered inadequate if started at >16 weeks and/or comprising fewer than two visits [52]. In addition to the self-reported history of illness during pregnancy, data on HIV status, urine analysis, Rhesus factor, stool examination, venereal diseases, hepatitis B, hemoglobin, and other illnesses were retrieved from prenatal records when available. Based on the measurement at prenatal care booking, prenatal anemia was defined as hemoglobin <11 g/dL.

#### Perinatal distress and birth outcome measures

Distress was assessed at inclusion, i.e., at or before 20 weeks of gestation and at 32 to 36 weeks of gestation. As the “perinatal period” often refers to the period from 22 weeks of gestation up to the end of the first week post-partum, we distinguish perinatal distress, referring to the measure of distress at 32 to 36 weeks of gestation, from early antenatal distress measured at inclusion. Anxiety was measured using the seven-item anxiety subscale of the Hospital Anxiety and Depression Scale (HADS-A), each item rated from 0–3. The HADS-A score was dichotomized at ≥8, a cut-off associated with clinically significant anxiety symptoms [53]. Depression was measured using the ten-item Edinburgh Postnatal Depression Scale (EPDS), each item rated from 0 to 3. A total EPDS score of ≥13 was defined as high depressive symptoms [54]. For stress, the four-item Perceived Stress Scale (PSS-4) was used, with each item rated from 0 to 4. A total score ≥8 was defined as high symptoms of stress [55].

Overall distress was defined as high symptoms in at least one of the three domains of distress, i.e., anxiety, depression, or stress. In addition, presence of high symptoms in one, two, or three domains were considered to indicate the level of distress. Likewise, overall distress over time and level of distress over time were generated to show the change during pregnancy from inclusion to 32 to 36 weeks of gestation. As a continuous outcome, a total distress score was obtained by summing the standardized anxiety, depression, and stress scores. Finally, a change in the scores of each measure of distress over the course of pregnancy was also computed by subtracting the scores at inclusion from the corresponding values at 32 to 36 weeks of gestation.

The internal consistency of the scales was assessed using Cronbach’s alpha, which yielded 0.87 for the anxiety scale, 0.71 for perceived stress, and 0.78 for depression at inclusion. At 32 to 36 weeks of gestation, the internal consistency remained almost same for the anxiety and depression scales while it increased to 0.77 for perceived stress.

Regarding adverse birth outcomes, gestational age was estimated from self-reported last menstrual period, fundal palpation, and/or ultrasound. The latter two were extracted from antenatal records. Preterm birth (PTB) was defined as gestational age <37 completed weeks. Birth weight was either retrieved from delivery records or measured within three days after birth for those born at home. Weight <2.5 kg at birth was classified as low birth weight (LBW) and weight below the 10^th^ percentile for gestational age at birth, and sex was classified as small for gestational age (SGA) according to international standards proposed by the INTERGROWTH-21^st^ project [56].

#### Statistical analyses

Characteristics of the participating women were summarized using proportions, means with standard deviations (SD), or medians with inter-quartile range (IQR). Chi-squared tests, T-test, or Mann-Whitney-U tests were used as appropriate to compare the distributions of socioeconomic adversity, distress at inclusion, the change in distress over the duration of pregnancy (i.e., from inclusion to the perinatal period), and perinatal distress between groups according to birth outcomes. P-values <0.05, tested two-sided, were considered statistically significant.

To evaluate the influence of each measures of perinatal distress on birth outcomes, logistic regression with a sparse modeling approach was utilized. That is, socioeconomic, reproductive, obstetric, and nutritional confounders that were significantly associated with adverse birth outcomes in univariable logistic regression analyses were included individually in models of each perinatal distress measure that had a significant association with a birth outcome measure. Furthermore, once each odds ratio was determined for the models with one confounder included, confounders were only considered relevant for the final models if the odds ratios corresponding to the perinatal distress measure changed by more than 10%, compared to the unadjusted models. A similar approach was applied to assess the influence of the change in each measure of distress over the span of pregnancy on adverse birth outcomes, as well as the associations between socioeconomic adversity and adverse birth outcomes. As we measured several variables that are related to each other, the sparse modeling approach was chosen so as to include only relevant variables in the final model(s).

When logistic regression showed an association between measures of perinatal distress and adverse birth outcomes, we applied a mediation analyses to examine whether perinatal distress is a mediator in the pathway between socioeconomic adversity and the adverse birth outcomes. The mediation analyses were done using the mediate function in R [57]. For each socioeconomic adversity variable significantly associated with perinatal distress in the univariable analysis, confounders that changed the unadjusted effect on the mediator and/or the respective outcome by more than 10% were included in the respective mediation model. Additionally, interaction between each indicator of the socioeconomic adversity and mediator was checked and included in the analyses when appropriate. The mediators were analyzed as a continuous and the outcome variables as a binary.

Furthermore, we conducted sensitivity analyses for the mediation effects using the medsens function in R to quantify the degree of violation of sequential ignorability assumption due to the presence of unmeasured confounders. The results of the sensitivity analyses are presented as correlated error terms between the error in the mediator model and the error in the outcome models. Stata (Version 14 SE, Stata Corporation, and College Station, Texas, USA) was used for all other analyses.

*Ethics statement*. The study protocol [(ref. number: IRB 026/2017 dated 15/08/2017)] was approved by the Institutional Research Review Board of College of Health Science, Aksum University, Ethiopia. Verbal consent was obtained from each participant prior to data collection.

*Inclusivity in global research*. Additional information regarding the ethical, cultural, and scientific considerations specific to inclusivity in global research is included in the S1 Checklist.

## Results

In total, 934 of the 991 included women were followed until delivery and had completed measures of distress with at least one known birth outcome (S1 Fig). The characteristics of the women with incomplete data who were excluded from the final analyses did not differ significantly from the women included in the analyses (S1 Table). The mean age of the women at inclusion was 29.3 years (SD = 6.5), and 289 (30.9%) received secondary education or above. Most women were farmers (54.6%), followed by housewives (34.2%), and most of them perceived their work as burdensome (59.0%). Furthermore, 72 (7.5%) had low social support. In reference to their reproductive and obstetric characteristics, the mean parity was 3.6 (SD = 2.3). With 379 (40.9%) of the index pregnancies being unplanned, nearly 55.0% of the women did not have adequate prenatal care. Also, 361 (38.7%) had a problem with food access, and 335 (35.9%) were underweight prior to the index pregnancy (Table 1).

**Table 1:** Selected characteristics of the participating women, overall and by birth outcome.

Socio-economic characteristics	Total, n = 934	PTB, n = 146	p-value	LBW, n = 147	p-value	SGA, n = 187	p-value
Age at inclusion, mean (SD)	29.3 (6.5)	30.1 (6.6)	.099	29.0 (6.1)	.484	28.7 (6.1)	.159
Rural residence, n (%)	605 (64.8)	106 (72.6)	.031	88 (59.9)	.170	114 (61.0)	.249
Orthodox Christians in religion, n (%)	922 (98.7%)	146 (100.0)	.621^d^	146 (99.3)	.217^d^	185 (98.9)	.100^d^
Educational status, n (%)			.212^e^		.324^e^		.538^e^
No formal education	338 (36.2)	62 (42.5)		52 (35.4)		67 (35.8)	
Primary education	307 (32.9)	41 (28.1)		42 (28.6)		57 (30.5)	
Secondary education or above	289 (30.9)	43 (29.5)		53 (36.1)		63 (33.7)	
Occupation, n (%)			.438		.443		.746
Farmer	506 (54.1)	86 (58.9)		73 (49.7)		98 (52.4)	
Housewife	321 (34.4)	44 (30.1)		57 (38.8)		69 (36.9)	
Others^a^	107 (11.5)	16 (11.0)		17 (11.5)		20 (10.7)	
Household size including the newborn, mean (SD)	5.5 (2.0)	4.7 (2.2)	.066	4.6 (2.1)	.562	4.5 (2.1)	.887
Quintiles of wealth index, n (%)			.016		.782		.819
Lowest	189 (20.2)	39 (26.7)		31 (21.1)		33 (17.7)	
Low	185 (19.8)	23 (15.8)		24 (16.3)		37 (19.8)	
Middle	190 (20.4)	21 (14.4)		30 (20.4)		41 (21.9)	
High	186 (19.9)	25 (17.1)		33 (22.5)		41 (21.9)	
Highest	184 (19.7)	38 (26.0)		29 (19.7)		35 (18.7)	
Model household, n (%)	229 (24.5)	29 (18.9)	.187	24 (16.3)	.021	32 (17.1)	.016
History of pre-pregnancy illness, n (%)	128 (13.7)	31 (21.2)	.693	41 (27.9)	.073	52 (27.8)	.017
History of chronic non-communicable diseases^b^, n (%)	15 (1.6)	2 (1.4)	.576	1 (0.7)	.281	2 (1.1)	.385
Perceived work burden, n (%)			.008		.442		.375
Easy	383 (41.0)	51 (34.9)		53 (36.1)		66 (35.3)	
Moderate	414 (44.3)	33 (22.6)		20 (13.6)		24 (12.8)	
Difficult	137 (14.7)	62 (42.5)		74 (50.3)		97 (51.9)	
Total social support score, mean (SD)	21.3 (3.8)	20.9 (3.5)	.243	20.1 (4.1)	.000	20.1 (4.0)	.000
Low total social support, n (%)	72 (7.7)	11 (7.5)		23 (15.7)		27 (14.4)	
At least one stressful life events, n (%)	343 (36.7)	283 (82.5)	.233	279 (82.3)	.272	266 (78.5)	.479
**Reproductive and obstetric conditions**							
Parity including index birth outcome, mean (SD)	3.6 (2.3)	3.9 (2.4)	.086	3.6 (2.2)	.990	3.6 (2.2)	.633
History of adverse birth outcome, n (%)	187 (20.0)	29 (19.9)	.100	26 (17.7)	.390	39 (20.9)	.914
Unplanned index pregnancy, n (%)	379 (40.6)	77 (53.0)	.002	63 (42.9)	.585	72 (38.5)	.522
Intimate partner violence score, mean (SD)	6.9 (3.0)	7.0 (3.2)	.823	7.7 (3.4)	.001	7.9 (3.3)	.000
Intimate partner violence, n (%)	151 (16.2)	28 (19.2)		39 (26.5)		53 (28.3)	
Women empowerment score, mean (SD)	5.6 (1.5)	5.5 (1.5)	.412	5.1 (1.3)	.000	5.2 (1.4)	.000
Empowered women, n (%)	104 (11.3)	16 (11.0)		8 (5.4)		12 (6.4)	
Adequacy of prenatal care, n (%)			.267		.046^e^		.042^e^
Inadequate	385 (41.2)	67 (45.9)		72 (49.0)		90 (48.1)	
Intermediate	112 (12.0)	20 (13.7)		16 (10.9)		20 (10.7)	
Adequate or adequate plus	437 (46.7)	59 (40.4)		59 (40.3)		77 (41.1)	
History of illness during pregnancy^c^, n (%)	210 (22.5)	31 (21.2)	.693	41 (27.9)	.073	52 (27.8)	.017
Al least one pregnancy complication, n (%)	506 (54.2)	73 (50.0)	.271	82 (55.8)	.692	102 (54.6)	.937
Hypertensive at 32–36 weeks, n (%)	57 (6.1)	11 (7.5)	.431	15 (10.2)	.033	18 (9.6)	.031
Negative Rhesus factor, n (%)	23 (2.5)	2 (1.4)	.560	2 (1.4)	.556	5 (2.7)	.784
**Nutritional characteristics**							
Food insecurity score, median (IQR)	0 (0–8)	0 (0–9)	.195^f^	0 (0–10)	.001^f^	0 (0–10)	.000^f^
Food insecure, n (%)	361 (38.7)	61 (41.8)		70 (47.6)		92 (49.2)	
Dietary diversity score, mean (SD)	4.6 (1.4)	4.5 (1.4)	.283	4.5 (1.3)	.246	4.4 (1.3)	.077
Adequate dietary diversity, n (%)	336 (36)	68 (46.6)		70 (47.6)		86 (46.0)	
Fasting, n (%)	650 (69.6)	117 (80.1)	.004	110 (74.8)	.140	14 (75.9)	.047
Alcohol intake at least once per week, n (%)	221 (23.7)	35 (24.0)	.638	31 (21.1)	.403	45 (24.1)	.995
Coffee intake per day, mean number of times (SD)	1.5 (1.0)	1.5 (0.1)	.742	1.4 (0.1)	.455	1.4 (0.1)	.673
Height in cm, mean (SD)	157.4 (0.06)	157.5 (0.06)	.935	157.2 (0.06)	.594	157.1 (0.06)	.517
Pre-pregnancy BMI kg/m^2^, mean (SD)	19.7 (2.0)	19.3 (2.1)	.009	18.8 (2.1)	.000	18.8 (1.9)	.000
Pre-pregnancy BMI <18.5 kg/m^2^, n (%)	335 (35.9)	69 (47.3)		89 (60.5)		110 (58.8)	
Hemoglobin in g/dL, mean (SD)	11.9 (1.6)	10.9 (0.12)	.000	10.4 (0.12)	.000	10.6 (0.11)	.000
Hemoglobin <11 g/dL, n (%)	271 (30.7)	58 (40.6)		39 (26.5)		62 (33.7)	
Gestational weight gain in kg, mean (SD)	10.6 (2.3)	9.0 (1.8)	.000	8.7 (1.7)	.000	8.8 (1.7)	.000
Inadequate gestational weight gain, n (%)	598 (64.0)	135 (92.5)		138 (93.9)		174 (93.1)	

^a^Students, unemployed, and so on,

^b^Consists of diabetes, hypertension and distress,

^c^includes diarrheal diseases, malaria, HIV, other venereal diseases, hepatitis, and others,

^d^Fisher’s exact test,

^e^chi-square test for trend, and

^f^Mann-Whitney-U tests.

PTB, preterm birth; LBW, low birth weight; and SGA, small for gestational age. P-values indicate the difference in distribution between PTB versus non-PTB, LBW versus non-LBW, and SGA versus non-SGA by the respective characteristics of the women.

As seen in Table 2, the mean score (SD) for perinatal anxiety was 4.9 (3.4), for depression 8.0 (4.6), for stress 6.1 (2.8), and for total distress 19.0 (9.2). As to the change of each distress measure scores from inclusion to the perinatal period, the changes were only significant for anxiety and stress (mean difference for anxiety, 0.13 [95% CI: 0.07, 0.19]; for stress, -0.26 [-0.34, -0.19]; for depression, 0.03 [-0.03, 0.09]; and for total distress, -0.11 [-0.23, 0.01], data not shown). Overall, a high prevalence of distress was observed at both time points: at inclusion (≤20 weeks of gestation) and in the perinatal period (32–36 weeks of gestation). Specific to perinatal distress, 21.4% of the women had high symptoms in one, 12.5% in two, and 9.2% in three of the domains.

**Table 2:** Distribution of adverse birth outcome by distress measures over time during pregnancy.

Distress at inclusion (at ≤20 weeks)	Total, n = 934	PTB, n = 146	p-value	LBW, n = 147	p-value	SGA, n = 187	p-value
Anxiety score at inclusion, mean (SD)	4.8 (3.8)	5.1 (4.0)	.209	5.6 (4.2)	.004	5.7 (4.2)	.001
Stress score at inclusion, mean (SD)	6.4 (2.7)	6.7 (2.6)	.064	7.1 (2.8)	.000	7.1 (2.8)	.000
Depression score at inclusion, mean (SD)	8.0 (4.7)	8.3 (4.9)	.335	9.1 (5.0)	.002	9.1 (4.9)	.001
Total distress score at inclusion, mean (SD)	19.1 (9.7)	20.2 (10.0)	.137	21.8 (10.5)	.000	21.8 (10.4)	.000
Level of distress at inclusion, n (%)			.598		.004		.001
Not distressed at all	518 (55.5)	77 (52.8)		66 (44.9)		89 (47.6)	
Distressed in one domain	206 (22.1)	33 (22.6)		33 (22.5)		38 (20.3)	
Distressed in two domains	122 (13.1)	18 (12.3)		24 (16.3)		29 (15.5)	
Distressed in three domains	88 (9.4)	18 (12.3)		24 (16.3)		31 (16.6)	
**Perinatal distress (distress at 32 to 36 weeks)**							
Perinatal anxiety score, mean (SD)	4.9 (3.4)	5.2 (3.7)	.228	5.7 (3.8)	.003	5.7 (3.8)	.000
Perinatal stress score, mean (SD)	6.1 (2.8)	6.5 (2.9)	.055^£^	7.0 (2.9)	.000	6.9 (2.9)	.000
Perinatal depression score, mean (SD)	8.0 (4.6)	8.5 (4.9)	.181	9.2 (4.9)	.001	9.2 (4.9)	.000
Total perinatal distress score, mean (SD)	19.0 (9.2)	20.2 (9.8)	.085	21.8 (10.1)	.000	21.8 (10.0)	.000
Level of perinatal distress, n (%)			.351		.001		.000
Not distressed at all	531 (56.9)	77 (52.8)		69 (46.9)		89 (47.6)	
Distressed in one domain	200 (21.4)	31 (21.2)		31 (21.1)		42 (22.5)	
Distressed in two domains	117 (12.5)	19 (13.0)		21 (14.3)		24 (12.8)	
Distressed in three domains	86 (9.2)	19 (13.0)		26 (17.7)		32 (17.1)	
**Distress over time during pregnancy**							
Change in anxiety score, mean (SD)	0.13 (1.0)	0.1 (0.9)	.512	0.1 (0.9)	.442	0.1 (0.9)	.329
Change in stress score, mean (SD	-0.26 (1.2)	-0.2 (1.1)	.669	-0.2 (1.1)	.239	-0.2 (1.0)	.190
Change in depression score, mean (SD)	0.03 (1.0)	0.2 (0.7)	.087	0.10 (0.7)	.301	0.1 (0.7)	.269
Change in total distress score, mean (SD)	-0.11 (1.9)	0.01 (1.6)	.428	0.02 (1.5)	.395	0.01 (1.5)	.389
Overall distress over time, n (%)			.427*		.010*		.020*
Not distressed at all	493 (52.8)	74 (50.7)		65 (44.2)		85 (45.5)	
Distressed only at inclusion	38 (4.1)	3 (2.1)		4 (2.7)		4 (2.1)	
Distressed only at 32 to 36 weeks	25 (2.7)	3 (2.1)		1 (0.7)		4 (2.1)	
Distressed at both time points	378 (40.5)	66 (45.2)		77 (52.4)		94 (50.3)	
Level of distress over time, n (%)			.318*		.037*		.030*
Not distressed at all	493 (52.8)	74 (50.7)		65 (44.2)		85 (45.5)	
Decreased level of distress	65 (7.0)	6 (4.1)		8 (5.4)		10 (5.4)	
Remained distressed with no change	331 (35.4)	57 (39.0)		68 (46.3)		84 (44.9)	
Increased level of distress	45 (4.8)	9 (6.2)		6 (4.1)		8 (4.3)	

*Fishers exact test, and

^£^Mann-Whitney-U tests.

PTB, preterm birth; LBW, low birth weight; and SGA, small for gestational age.

**Fig 1:**
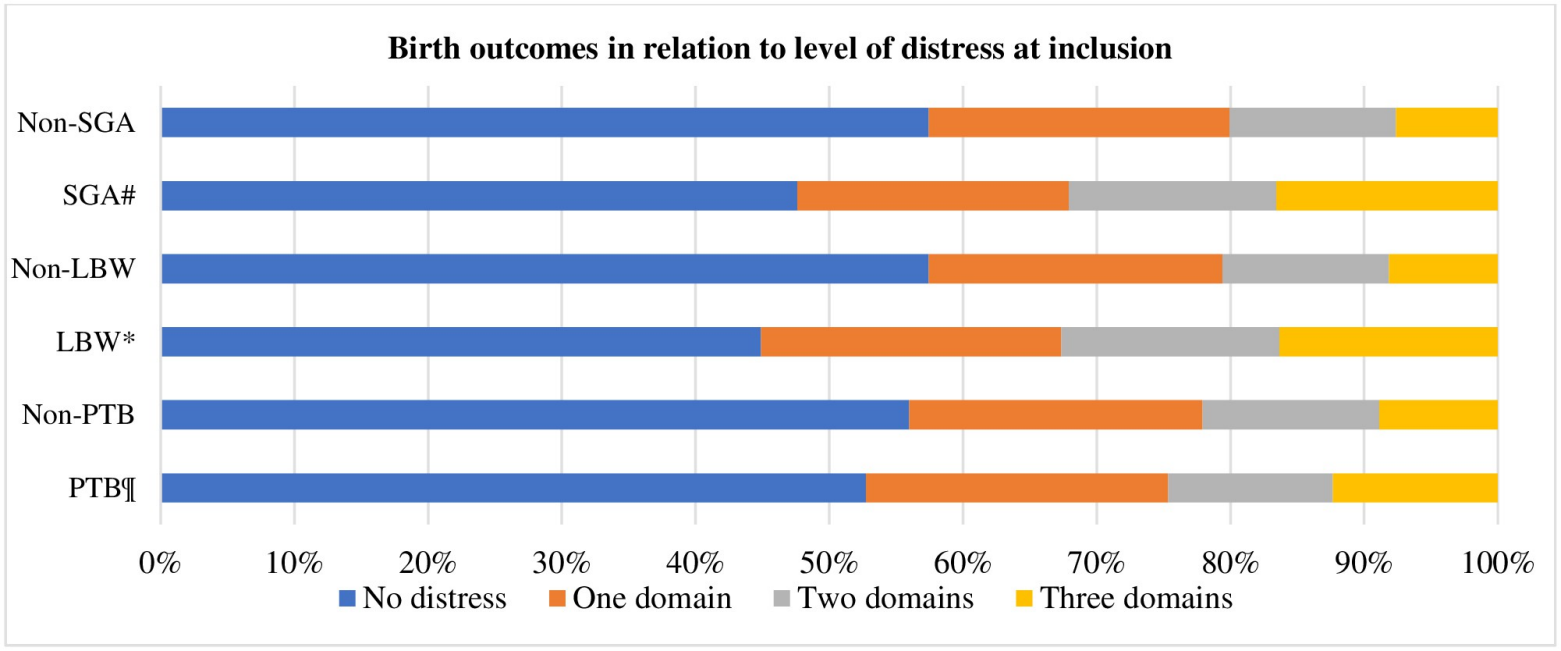
Birth outcomes in relation to level of distress at inclusion. ^¶^p-value = 0.598 PTB versus non-PTB, *p-value = 0.004 LBW versus non-LBW, and ^#^p-value = 0.001 SGA versus non-SGA.

**Fig 2:**
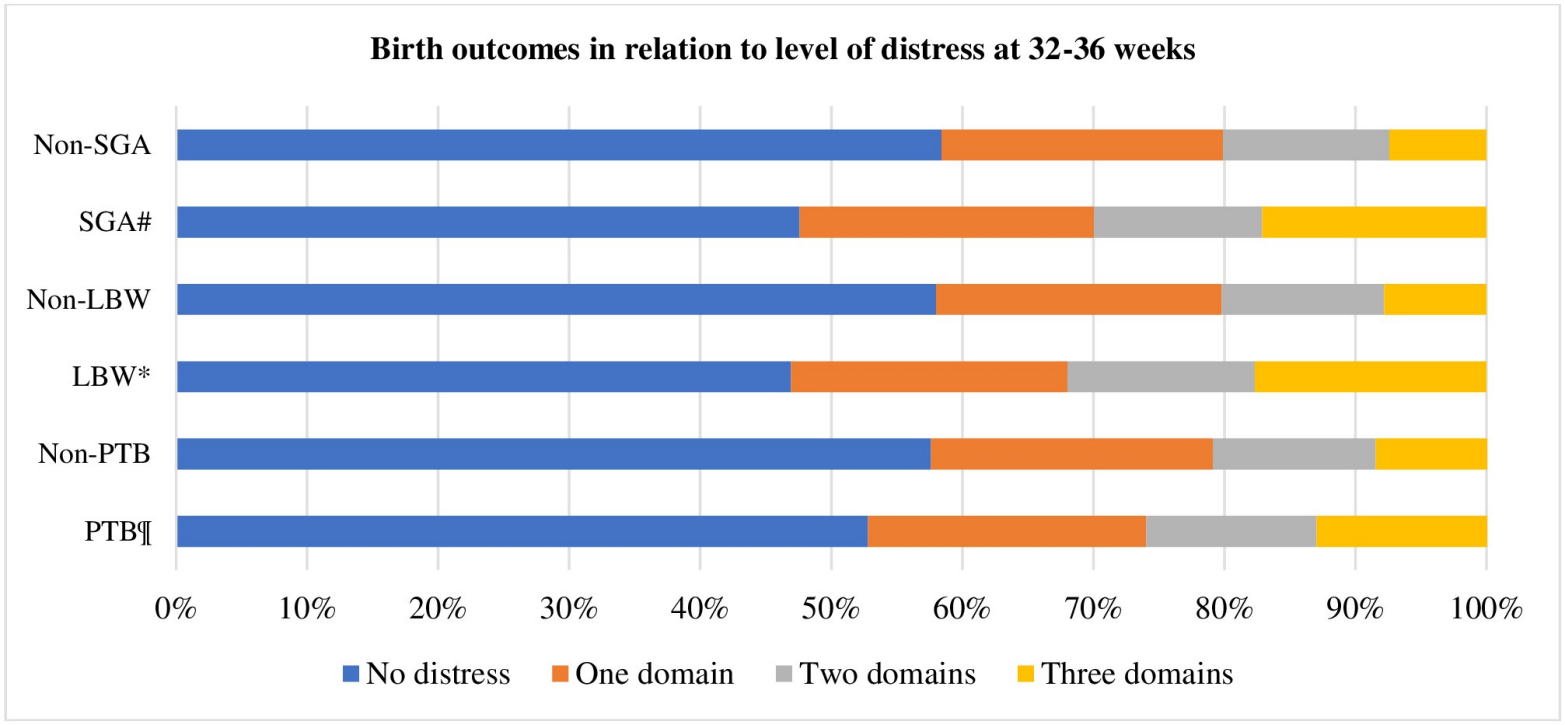
Birth outcomes in relation to level of distress at 32 to 36 weeks of gestation. ^¶^p = 0.351 PTB versus non-PTB,*p = 0.001 LBW versus non-LBW, and ^#^p = 0.000 SGA versus non-SGA.

All continuous measures of perinatal distress—anxiety, depression, stress, and total distress—were associated with low birth weight and small for gestational age, while none of them did with preterm birth. Of the change in measures of distress over time, none of the changes in measures of distress over time was associated with the adverse birth outcomes (Table 2). Additionally, level of perinatal distress was associated with low birth weight and small for gestational age but not with preterm birth as were overall distress and level of distress over time during pregnancy (Figs 1 and 2).

In the logistic regression models, perinatal anxiety (OR [95% CI] 1.08 [1.02, 1.13]), stress (1.14 [1.07, 1.22]), depression (1.07 [1.03, 1.11]), and total distress (1.15 [1.07, 1.23]) were significantly associated with higher odds of low birth weight, and small for gestational age. None of the measures of perinatal distress was associated with preterm birth. Also, level of perinatal distress was not associated with any of the adverse birth outcomes. Moreover, overall distress and level of distress over time during the course of pregnancy were not associated with any adverse outcome. From the socioeconomic adversities, only intimate partner violence was associated with small for gestational age (Table 3).

**Table 3:** Associations of measures of distress and socioeconomic adversities with birth size.

Measures of distress	LBW				SGA			
	COR (95% CI)	p-value	AOR (95% CI)	p-value	COR (95% CI)	p-value	AOR (95% CI)	p-value
Perinatal anxiety score^1^	1.08 (1.02, 1.13)	.004	1.08 (1.02, 1.13)	**.004**	1.08 (1.04, 1.13)	.000	1.08 (1.04, 1.13)	**.000**
High symptoms of perinatal anxiety^2^	1.48 (1.00, 2.20)	.051	Not applicable	**-**	1.53 (1.07, 2.20)	.020	0.91 (0.57, 1.45)	.678
Perinatal stress score^1^	1.14 (1.07, 1.22)	.000	1.14 (1.07, 1.22)	**.000**	1.14 (1.07, 1.21)	.000	1.14 (1.07, 1.21)	**.000**
High symptoms of perinatal stress^2^	2.02 (1.41, 2.90)	.000	1.29 (0.80, 1.92)	.338	2.02 (1.45, 2.82)	.000	1.18 (0.77, 1.79)	.449
Perinatal depression score^1^	1.07 (1.03, 1.11)	.001	1.07 (1.03, 1.11)	**.001**	1.07 (1.04, 1.11)	.000	1.07 (1.04, 1.11)	**.000**
High symptoms of perinatal depression^2^	1.78 (1.20, 2.64)	.004	1.19 (0.74, 1.91)	.469	1.55 (1.07, 2.24)	.020	0.87 (0.55, 1.37)	.551
Total perinatal distress score^1^	1.15 (1.07, 1.23)	.000	1.15 (1.07, 1.23)	**.000**	1.16 (1.09, 1.23)	.000	1.16 (1.09, 1.23)	**.000**
Level of perinatal distress^2^								
Not distressed at all	Reference	-	Reference	-	Reference	-	Reference	-
Distressed in one domain	1.23 (0.78, 1.95)	.380	0.95 (0.57, 1.57)	.830	1.32 (0.88, 1.99)	.183	0.94 (0.59, 1.50)	.794
Distressed in two domains	1.46 (0.85, 2.49)	.171	0.92 (0.47, 1.83)	.815	1.27 (0.77, 2.11)	.223	0.66 (0.34, 1.28)	.223
Distressed in three domains	2.90 (1.71, 4.91)	.000	1.36 (0.64, 2.91)	.430	2.94 (1.80, 4.83)	.000	1.10 (0.55, 2.18)	.790
Overall distress over time^2^								
Not distressed at all	Reference	-	Reference	-	Reference	-	Reference	-
Distressed only at inclusion	0.79 (0.27, 2.29)	.658	0.91 (0.24, 3.41)	.885	0.57 (0.20, 1.66)	.303	0.61 (0.17, 2.27)	.463
Distressed only at 32 to 36 weeks	0.28 (0.04, 2.12)	.219	0.19 (0.02, 1.45)	.186	0.94 (0.31, 2.83)	.917	0.60 (0.18, 1.94)	.389
Distressed at both time points	1.68 (1.17, 2.41)	.005	1.06 (0.65, 1.71)	.823	1.58 (1.14, 2.21)	.006	0.87 (0.56, 1.35)	.528
Level of distress over time^2^								
Not distressed at all	Reference	-	Reference	-	Reference	-	Reference	-
Decreased level of distress	0.93 (0.42, 2.03)	.846	0.97 (0.38, 2.48)	.947	0.87 (0.43, 1.79)	.711	0.86 (0.37, 2.00)	.732
Remained distressed with no change	1.70 (1.17, 2.47)	.005	1.03 (0.63, 1.69)	.895	1.63 (1.16, 2.29)	.005	0.87 (0.55, 1.37)	.541
Increased level of distress	1.02 (0.42, 2.52)	.961	0.57 (0.63, 1.69)	.239	1.05 (0.47, 2.34)	.908	0.50 (0.21, 1.17)	.108
**Socioeconomic adversities**								
Intimate partner violence, yes^3^	2.16 (1.42, 3.28)	.000	1.26 (0.75, 2.13)	.381	2.60 (1.77, 3.81)	.000	1.66 (1.03, 2.68)	**.038**
Low social support, yes^3^	3.20 (1.84, 5.59)	.000	1.70 (0.81, 3.60)	.163	3.25 (1.91, 5.52)	.000	1.66 (0.83, 3.21)	.160
Food insecure, yes^3^	1.54 (1.08, 2.20)	.017	1.00 (0.96, 1.05)	.869	1.71 (1.24, 2.37)	.001	1.02 (0.98, 1.05)	.451
Not empowered women, yes^3^	2.46 (1.17, 5.18)	.018	1.57 (0.71, 3.45)	.267	2.09 (1.12, 3.90)	.021	1.31 (0.88, 1.95)	.435
At least one stressful life events, yes^2^	1.22 (0.85, 1.75)	.272	0.96 (0.63, 1.47)	.854	1.13 (0.81, 1.57)	.479	0.80 (0.54, 1.19)	.277

^1^none of the covariates altered the unadjusted odds ratios by more than 10% and the unadjusted models are presented as the final models.

^2^adjusted for pre-pregnancy BMI, gestational weight gain, social support, women empowerment, intimate partner violence, and food insecurity.

^3^adjusted for pre-pregnancy BMI, gestational weight gain, intimate partner violence, social support, food insecurity, women empowerment, and perinatal distress.

Table 4 presents the results of the mediation analyses assessing whether perinatal distress is a mediator in the pathway between socioeconomic adversity and adverse birth outcomes. Most socioeconomic adversities were indirectly associated with the adverse birth outcomes through total perinatal distress score, showing that perinatal distress is a mediator. Similar findings were obtained with the individual measures of distress as mediators (S2–S4 Tables). Our sensitivity analyses showed that an omitted confounder must explain 20 to 30% of the remaining variance in the mediator (total perinatal distress score) and 20 to 30% of the remaining variance in the outcome (small birth size) for the average causal mediated effect to be zero.

**Table 4:** Results of mediation analysis assessing if perinatal distress is a mediator in the pathway between socioeconomic adversity and adverse birth outcome.

**For LBW as adverse birth outcome and total perinatal distress score as a mediator**	**Average direct effect**	**p-value**	**Average causal mediated effect**	**p-value**	**Total effect**	**p-value**	**Proportion mediated**
	**Coefficient (95% CI)**		**Coefficient (95% CI)**		**Coefficient (95% CI)**		
Wealth index	-0.009 (-0.078, 0.070)	.766		**.006**	-0.001 (-0.067, 0.080)		
Lowest	-0.040 (-0.104, 0.040)	.270	0.009 (0.002, 0.020)	.058	-0.033 (-0.096, 0.050)	.940	5.8%
Low	-0.009 (-0.076, 0.070)	.760	0.006 (-0.001, 0.0109)	.260	-0.005 (-0.071, 0.070)	.348	8.3%
Middle	0.011 (-0.060, 0.100)	.800	0.004 (-0.003, 0.010)	.780	0.012 (-0.060, 0.100)	.840	1.2%
High	Reference	-	0.001 (-0.007, 0.010)	-	Reference	.800	2.0%
Highest	0.078 (-0.001, 0.130)	.066	Reference	.792	0.079 (-0.009, 0.130)	**-**	
Not empowered women, yes	0.022 (-0.034, 0.080)	.410	0.001 (-0.006, 0.010)	**.000**	0.038 (-0.011, 0.09)	.064	1.3%
Food insecurity, yes	0.070 (-0.002, 0.015)	.062	0.018 (0.008, 0.030)	**.008**	0.091 (0.028, 0.160)	.120	42.6%
Intimate partner violence, yes	0.115 (0.012, 0.250)	**.032**	0.029 (-0.009, 0.050)	**.008**	0.153 (0.057, 0.280)	**.000**	33.5%
Low social support, yes	-0.001 (-0.049, 0.050)	.950	0.059 (0.020, 0.100)	**.016**	0.005 (-0.043, 0.050)	**.000**	40.5%
At least one stressful life event, yes	-0.009 (-0.078, 0.070)	.766	0.007 (0.001, 0.010)	**.006**	-0.001 (-0.067, 0.080)	.828	14.2%
**For SGA as adverse birth outcome and total perinatal distress score as a mediator**	**Average direct effect**	**p-value**	**Average causal mediated effect**	**p-value**	**Total effect**	**p-value**	**Proportion mediated**
	**Coefficient (95% CI)**		**Coefficient (95% CI)**		**Coefficient (95% CI)**		
Wealth index	-0.038 (-0.116, 0.050)	.362	0.011 (0.003, 0.020)	**.006**	-0.026 (-0.101, 0.060)	.516	
Lowest	-0.009 (-0.085, 0.090)	.754	0.008 (-0.001, 0.020)	.076	-0.001 (-0.077, 0.100)	.934	14.1%
Low	0.013 (-0.069, 0.110)	.810	0.005 (-0.004, 0.020)	.300	0.017 (-0.065, 0.110)	.740	2.8%
Middle	0.027 (-0.056, 0.130)	.620	0.001 (-0.009, 0.010)	.800	0.028 (-0.055, 0.130)	.580	5.1%
High	Reference	-	Reference	-	Reference	-	1.4%
Highest	0.080 (-0.008, 0.150)	.082	0.001 (-0.008, 0.010)	.756	0.081 (-0.005, 0.150)	.072	
Not empowered women, yes	0.045 (-0.013, 0.100)	.104	0.022 (0.010, 0.040)	**.000**	0.063 (0.009, 0.120)	**.022**	1.5%
Food insecurity, yes	0.126 (0.040, 0.22)	**.002**	0.034 (0.011, 0.06)	**.004**	0.146 (0.071, 0.240)	**.000**	34.8%
Intimate partner violence, yes	0.110 (-0.001, 0.230)	.052	0.073 (0.036, 0.120)	**.000**	0.161 (0.063, 0.280)	**.000**	23.4%
Low social support, yes	-0.013 (-0.068, 0.040)	.628	0.008 (0.002, 0.020)	**.014**	-0.004 (-0.057, 0.050)	.874	45.4%
At least one stressful life event, yes	-0.038 (-0.116, 0.050)	.362	0.011 (0.003, 0.020)	**.006**	-0.026 (-0.101, 0.060)	.516	12.2%

## Discussion

In the present study, we have investigated the influence of different measures of perinatal distress on adverse birth outcomes. We determined that perinatal distress was associated with adverse birth outcomes in this cohort of primarily rural Ethiopian women. Indeed, perinatal distress was a mediator in the pathway between socioeconomic adversity and adverse birth outcomes. Given the negative health effects of the adverse birth outcomes in early childhood, in later life, and future generations [22, 25], our findings may imply the importance of good mental health and adequate mental healthcare during pregnancy in low-income settings like Ethiopia. The results may also suggest an opportunity for a perinatal intervention to improve mental health and help halt the observed cycle of undernourishment being passed from mother to child via adverse birth outcomes and subsequent growth stunting.

All continuous measures of perinatal distress were associated with low birth weight and/or small for gestational age, consistent with several studies [58–62]. The association could be partly explained by the disruptions of the hypothalamic-pituitary-adrenocortical axis that restricts the oxygen and nutrients supply to the fetus. On the contrary, some studies in low-income countries, including the subgroup analysis of a recent meta-analysis, did not show an association between distress and low birth weight [25, 29, 35, 38, 63, 64]. The disagreement in results may stem from the difference in the modeling approach. A sparse modeling approach for confounding was applied in our study. However, the previous studies considered a large selection of confounders that were significant at p ≤0.2 in univariable models. Also, only categorical measures of distress were analyzed in the earlier studies that have implications on the study’s power. Notably, neither the individual nor the combined (level of perinatal distress) categorical measures of distress associate with low birth size in our study. Thus, the lack of association could be due to inadequate power.

Furthermore, perinatal distress was found to be a mediator in the pathway between socioeconomic adversity and low birth weight and/or small for gestational age. This finding implies that socioeconomically disadvantaged women will likely experience distress, leading to adverse birth outcomes. Therefore, our path analysis findings may highlight the importance of targeted screening and management of distress, focusing on women experiencing socioeconomic adversity. In low-income countries like Ethiopia, the screening and management of distress can be facilitated by integrating mental health better within primary maternal health care services.

Unlike birth size, we did not detect an influence by any measure of distress on preterm birth, which was consistent with birth-cohort studies in several low income countries, including the subgroup analyses of a recent meta-analysis [25, 35, 37, 64]. In contrast, there are recent meta-analyses where the different measures of perinatal distress appear to be clearly linked with preterm birth [58–60, 65]. The disagreement in the findings with some of the previous studies could be due to the difference in the definition of preterm birth. Preterm birth was defined as any live birth between ≥20 and <37 weeks of gestation by most of the previous studies. In low income countries like Ethiopia, the lower limit for viable birth is 28 weeks of gestation. Therefore, births at or after 20 weeks and before 28 weeks of gestation were not included in our analysis.

Interestingly, our data also showed that none of the changes in distress measures over time were associated with adverse birth outcomes. The difference in each measure of distress over time during pregnancy, however, was small. Thus, the insignificant change in scores might indicate persistent high distress and suggest the need for maternal mental health interventions starting from early pregnancy.

One of the major strengths of our study is that our data considered a broad range of distress measurements in both early and late pregnancy and their influence on adverse birth outcomes. However, our study is limited in that bio-specimens were not collected. Hence, we could not measure biomarkers such as cortisol or norepinephrine, validated markers of distress and could be seen as surrogate markers of possible changes in oxygen and nutrient supply to the fetus, linked to adverse birth outcomes. Also, the gestational age estimated by ultrasound was not available for most women, so it is possible that our small for gestational age data is not sufficiently accurate. Additionally, our study design was not suitable to rule out the probable reverse causality between distress and adverse birth outcomes due to the two-way conversation between the fetus and the mother. Finally, distress was assessed via an interviewer-administered questionnaire, and our data may be subjected to misclassification bias.

## Conclusions

Our study revealed that perinatal distress was linked with adverse birth outcomes and acted as a mediator between socioeconomic adversity and these outcomes. Our findings highlight the importance of screening women for distress and providing appropriate interventions, focusing on women experiencing socioeconomic adversity. Integrating mental health services into primary maternal care in low-income countries could be an effective approach to achieve this.

## Supporting information

S1 Fig Study flow chart. (DOCX)

S1 Table Baseline characteristics of women who were and were not in the complete follow-up sample, northern Ethiopia 2018. (DOCX)

S2 Table Results of mediation analysis assessing if perinatal distress is a mediator in the pathway between socioeconomic adversity and adverse birth outcome. (DOCX)

S3 Table Results of mediation analysis assessing if perinatal distress is a mediator in the pathway between socioeconomic adversity and adverse birth outcome. (DOCX)

S4 Table Results of mediation analysis assessing if perinatal distress is a mediator in the pathway between socioeconomic adversity and adverse birth outcome. (DOCX)

S1 Dataset KITE cohort minimal dataset. (DTA)

S1 Checklist Inclusivity in global research. (DOCX)

S2 Checklist STROBE statement—Checklist of items that should be included in reports of observational studies. (DOCX)
