# Supplementary figures and images for: Influence of perinatal distress on adverse birth outcomes: A prospective study in the Tigray region, northern Ethiopia
Source: PLoS One. 2023 Jul 13;18(7):e0287686. doi: 10.1371/journal.pone.0287686 (PMC10343148; doi:10.1371/journal.pone.0287686)

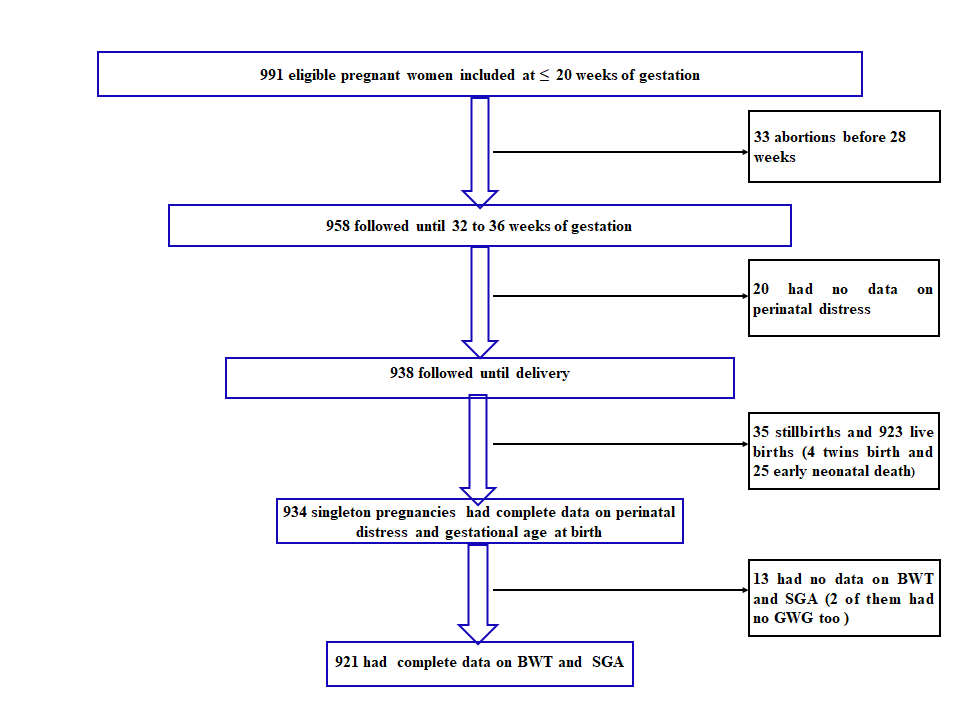


**S1 Fig. Study flow chart**

Supplement: S1 Fig — (DOCX) [file pone.0287686.s001.docx]
