# Supplementary material for: Influence of perinatal distress on adverse birth outcomes: A prospective study in the Tigray region, northern Ethiopia
Source: PLoS One. 2023 Jul 13;18(7):e0287686. doi: 10.1371/journal.pone.0287686 (PMC10343148; doi:10.1371/journal.pone.0287686)
Supplement: S1 Table — (DOCX) [file pone.0287686.s002.docx]

**S1 Table. Baseline characteristics of women who were and were not in the complete follow-up sample, northern Ethiopia 2018**

| **Characteristics** | **For PTB** | | | **For LBW and SGA** | | |
| --- | --- | --- | --- | --- | --- | --- |
|  | **Followed, n=934** | **Not followed, n=57** | ***P*-value** | **Followed, n=921** | **Not followed, n=70** | ***P*-value** |
| Age at inclusion, mean (SD) | 29.3 (6.5) | 29.4 (6.3) | .887 | 29.3 (6.5) | 29.3 (6.1) | .982 |
| Rural residence, n (%) | 605 (64.8) | 42 (73.7) | .170 | 597 (64.8) | 50 (71.4) | .263 |
| Educational status of woman, n (%) |  |  | .540 |  |  | .654 |
| No formal education | 338 (36.2) | 24 (42.1) |  | 335 (36.4) | 27 (38.6) |  |
| Primary education | 307 (32.9) | 19 (33.3) |  | 301 (32.7) | 25 (35.7) |  |
| Secondary education and above | 289 (30.9) | 14 (24.6) |  | 285 (30.9) | 18 (25.7) |  |
| Occupation of woman, n (%) |  |  | .554 |  |  | .404 |
| Farmer | 506 (54.1) | 35 (61.4) |  | 500 (54.3) | 41 (58.6) |  |
| Housewife | 321 (34.4) | 16 (28.1) |  | 318 (34.5) | 19 (27.1) |  |
| Others* | 107 (11.5) | 6 (10.5) |  | 103 (11.2) | 10 (14.3) |  |
| Family size, mean (SD) | 5.5 (2.0) | 5.8 (2.2) | .256 | 5.5 (2.0) | 5.7 (2.2) | .439 |
| Quintiles of wealth index, n (%) |  |  | .376 |  |  | .413 |
| Lowest | 189 (20.2) | 17 (29.8) |  | 184 (20.0) | 19 (27.1) |  |
| Low | 185 (19.8) | 10 (17.5) |  | 183 (19.9) | 13 (18.6) |  |
| Middle | 190 (20.4) | 8 (14.0) |  | 187 (20.2) | 10 (14.3) |  |
| High | 186 (19.9) | 11 (19.3) |  | 184 (20.0) | 16 (22.9) |  |
| Highest | 184 (19.7) | 11 (19.3) |  | 183 (19.9) | 12 (17.1) |  |
| Access to improved source of drinking water, n (%) | 835 (89.4) | 53 (93.0) | .390 | 823 (89.4) | 65 (92.9) | .355 |
| Time to fetch water not exceed 30 minutes, n (%) | 745 (79.8) | 43 (75.4) | .442 | 734 (79.7) | 54 (77.1) | .614 |
| Access to improved sanitation facility, n (%) | 132 (14.1) | 3 (5.3) | .058 | 129 (14.0) | 6 (8.6) | .201 |
| Model household, n (%) | 225 (25.5) | 13 (22.8) | .770 | 222 (24.1) | 20 (28.6) | .402 |
| Parity including the index birth, mean (SD) | 3.6 (2.3) | 3.9 (2.3) | .344 | 3.6 (2.3) | 3.9 (2.2) | .375 |
| MUAC at inclusion in cm, mean (SD) | 22.6 (2.0) | 22.3 (1.6) | .184 | 22.6 (2.0) | 22.3 (1.5) | .207 |
| Pre-pregnancy BMI in kg/m^2^_,_ mean (SD) | 19.73 (2.0) | 19.4 (1.6) | .220 | 19.73 (2.0) | 19.5 (1.6) | .231 |

*Students, unemployed, and so on. PTB; preterm birth, LBW; low birthweight, and SGA; small for gestational age.
