# Supplementary material for: Influence of perinatal distress on adverse birth outcomes: A prospective study in the Tigray region, northern Ethiopia
Source: PLoS One. 2023 Jul 13;18(7):e0287686. doi: 10.1371/journal.pone.0287686 (PMC10343148; doi:10.1371/journal.pone.0287686)
Supplement: S2 Table — (DOCX) [file pone.0287686.s003.docx]

| S2 Table. Results of mediation analysis assessing if perinatal distress is a mediator in the pathway between socioeconomic adversity and adverse birth outcome | | | | | | | |
| --- | --- | --- | --- | --- | --- | --- | --- |
| For LBW as adverse birth outcome and  total perinatal anxiety score as a mediator | **Average direct effect** | ***P*-value** | **Average causal mediated effect** | ***P*-value** | **Total effect** | ***P*-value** | **Proportion mediated** |
|  | **Coefficient (95% CI)** |  | **Coefficient (95% CI)** |  | **Coefficient (95% CI)** |  |  |
| Wealth index |  |  |  |  |  |  |  |
| Lowest | 0.002 (-0.070, 0.090) | .100 | 0.005 (-0.001, 0.020) | .110 | 0.007 (-0.063, 0.010) | .910 | 3.1% |
| Low | -0.032 (-0.097, 0.050) | .360 | 0.003 (-0.002, 0.010) | .270 | -0.029 (-0.093, 0.050) | .400 | 3.5% |
| Middle | -0.002 (-0.073, 0.080) | .900 | 0.004 (-0.002, 0.010) | .240 | 0.002 (-0.069, 0.090) | .970 | 0.1% |
| High | 0.021 (-0.056, 0.120) | .640 | -0.001 (-0.008, 0.010) | .780 | 0.021 (-0.058, 0.120) | .640 | 0.4% |
| Highest | Reference | **-** | Reference | **-** | Reference | **-** |  |
| Not empowered women, yes | 0.085 (0.011, 0.140) | **.022** | -0.001 (-0.008, 0.010) | .606 | 0.084 (0.010, 0.140) | **.024** | 1.5% |
| Food insecurity, yes | 0.039 (-0.012, 0.090) | .130 | 0.007 (0.001, 0.020) | **.020** | 0.045(-0.004, 0.100) | .070 | 15.5% |
| Intimate partner violence, yes | 0.096 (0.023, 0.190) | **.006** | 0.021 (-0.004, 0.050) | .080 | 0.109 (0.044, 0.190) | **.000** | 20.1% |
| Low social support, yes | 0.157 (0.037, 0.310) | **.012** | 0.050 (0.013, 0.090) | **.004** | 0.184 (0.074, 0.330) | **.002** | 27.9% |
| At least one stressful life event, yes | 0.012 (-0.042, 0.070) | .612 | 0.007 (0.002, 0.010) | **.002** | 0.019 (-0.035, 0.070) | .448 | 20.5% |
| For SGA as adverse birth outcome and  total perinatal anxiety score as a mediator | **Average direct effect** | ***P*-value** | **Average causal mediated effect** | ***P*-value** | **Total effect** | ***P*-value** | **Proportion mediated** |
|  | **Coefficient (95% CI)** |  | **Coefficient (95% CI)** |  | **Coefficient (95% CI)** |  |  |
| Wealth index |  |  |  |  |  |  |  |
| Lowest | -0.029 (-0.104, 0.060) | .476 | 0.007 (-0.001, 0.020) | .082 | -0.021 (-0.094, 0.060) | .586 | 8.1% |
| Low | 0.003 (-0.077, 0.010) | .100 | 0.004 (-0.004, 0.010) | .280 | 0.007 (-0.072, 0.110) | .910 | 2.9% |
| Middle | 0.017 (-0.061, 0.110) | .750 | 0.005 (-0.003, 0.020) | .220 | 0.022 (-0.055, 0.110) | .650 | 6.4% |
| High | 0.033 (-0.049, 0.120) | .440 | -0.002 (-0.011, 0.010) | .770 | 0.032 (-0.051, 0.120) | .470 | 1.3% |
| Highest | Reference | **-** | Reference | **-** | Reference | **-** |  |
| Not empowered women, yes | 0.088 (0.006, 0.150) | **.032** | -0.002 (-0.011, 0.010) | .582 | 0.087 (0.004, 0.150) | **.040** | 2.1% |
| Food insecurity, yes | 0.067 (0.009, 0.130) | **.026** | 0.010 (0.003, 0.020) | **.008** | 0.074 (0.017, 0.130) | **.014** | 12.6% |
| Intimate partner violence, yes | 0.153 (0.071, 0.250) | **.000** | 0.025 (-0.006, 0.050) | .082 | 0.168 (0.094, 0.260) | **.000** | 16.8% |
| Low social support, yes | 0.221 (0.086, 0.390) | **.000** | 0.035 (-0.005, 0.080) | .086 | 0.238 (0.112, 0.400) | **.000** | 14.8% |
| At least one stressful life event, yes | -0.003 (-0.058, 0.050) | .930 | 0.009 (0.003, 0.020) | **.000** | 0.007 (-0.047, 0.060) | .780 | 22.4% |
