# Supplementary material for: Influence of perinatal distress on adverse birth outcomes: A prospective study in the Tigray region, northern Ethiopia
Source: PLoS One. 2023 Jul 13;18(7):e0287686. doi: 10.1371/journal.pone.0287686 (PMC10343148; doi:10.1371/journal.pone.0287686)
Supplement: S3 Table — (DOCX) [file pone.0287686.s004.docx]

| S3 Table. Results of mediation analysis assessing if perinatal distress is a mediator in the pathway between socioeconomic adversity and adverse birth outcome | | | | | | | |
| --- | --- | --- | --- | --- | --- | --- | --- |
| For LBW as adverse birth outcome and total perinatal depression score as a mediator | **Average direct effect** | ***P*-value** | **Average causal mediated effect** | ***P*-value** | **Total effect** | ***P*-value** | **Proportion mediated** |
|  | **Coefficient (95% CI)** |  | **Coefficient (95% CI)** |  | **Coefficient (95% CI)** |  |  |
| Wealth index |  |  |  |  |  |  |  |
| Lowest | -0.004 (-0.074, 0.080) | .890 | 0.003 (-0.003, 0.010) | .310 | -0.001 (-0.066, 0.080) | .950 | 0.3% |
| Low | -0.035 (-0.100, 0.050) | .350 | 0.002 (-0.003, 0.010) | .460 | -0.032 (-0.095, 0.050) | .380 | 3.0% |
| Middle | 0.001 (-0.066, 0.080) | .950 | -0.0002 (-0.008, 0.010) | .950 | 0.001 (-0.067, 0.080) | .950 | 0.8% |
| High | 0.019 (-0.053, 0.110) | .690 | -0.002 (-0.010, 0.010) | .660 | 0.017 (-0.056, 0.110) | .730 | 1.5% |
| Highest | Reference | **-** | Reference | **-** | Reference | **-** |  |
| Not empowered women, yes | 0.082 (-0.004, 0.140) | .060 | 0.003 (-0.003, 0.010) | .338 | 0.083 (-0.003, 0.140) | .056 | 3.1% |
| Food insecurity, yes | 0.036 (-0.017, 0.090) | .170 | 0.012 (0.003, 0.020) | **.006** | 0.046 (-0.005, 0.090) | .072 | 24.5% |
| Intimate partner violence, yes | 0.090 (0.014, 0.180) | **.024** | 0.020 (-0.001, 0.040) | .056 | 0.104 (0.038, 0.190) | **.002** | 20.3% |
| Low social support, yes | 0.002 (-0.048, 0.050) | .954 | 0.005 (-0.0001, 0.010) | .064 | 0.007 (-0.042, 0.060) | .808 | 9.7% |
| At least one stressful life event, yes | 0.011 (-0.039, 0.070) | .684 | 0.008 (0.002, 0.010) | **.004** | 0.018 (-0.029, 0.070) | .514 | 20.0% |
| For SGA as adverse birth outcome and total perinatal depression score as a mediator | **Average direct effect** | ***P*-value** | **Average causal mediated effect** | ***P*-value** | **Total effect** | ***P*-value** | **Proportion mediated** |
|  | **Coefficient (95% CI)** |  | **Coefficient (95% CI)** |  | **Coefficient (95% CI)** |  |  |
| Wealth index |  |  |  |  |  |  |  |
| Lowest | -0.031 (-0.104, 0.060) | .430 | 0.004 (-0.003, 0.010) | .290 | -0.027 (-0.100, 0.070) | .500 | 4.3% |
| Low | -0.001 (-0.080, 0.010) | .960 | 0.003 (-0.005, 0.010) | .460 | 0.003 (-0.076, 0.100) | .100 | 0.5% |
| Middle | 0.013 (-0.069, 0.110) | .810 | 0.005 (-0.004, 0.020) | .300 | 0.017 (-0.065, 0.110) | .740 | 5.1% |
| High | 0.028 (-0.056, 0.120) | .560 | -0.002 (-0.012, 0.010) | .600 | 0.026 (-0.056, 0.120) | .590 | 1.9% |
| Highest | Reference | **-** | Reference | **-** | Reference | **-** |  |
| Not empowered women, yes | 0.085 (-0.009, 0.150) | .070 | 0.004 (-0.004, 0.010) | .352 | 0.087 (-0.004, 0.150) | .070 | 3.7% |
| Food insecurity, yes | 0.064 (0.004, 0.130) | **.028** | 0.014 (0.004, 0.003) | **.000** | 0.075 (0.019, 0.140) | **.008** | 18.9% |
| Intimate partner violence, yes | 0.147 (0.058, 0.240) | **.000** | 0.023 (0.001, 0.050) | **.044** | 0.161 (0.085, 0.250) | **.000** | 14.4% |
| Low social support, yes | 0.219 (0.080, 0.380) | **.002** | 0.045 (-0.014, 0.100) | .110 | 0.242 (0.118, 0.400) | **.000** | 19.4% |
| At least one stressful life event, yes | -0.001 (-0.057, 0.050) | .870 | 0.003 (-0.002, 0.010) | .270 | 0.002 (-0.051, 0.060) | .930 | 2.5% |
