# Supplementary material for: Influence of perinatal distress on adverse birth outcomes: A prospective study in the Tigray region, northern Ethiopia
Source: PLoS One. 2023 Jul 13;18(7):e0287686. doi: 10.1371/journal.pone.0287686 (PMC10343148; doi:10.1371/journal.pone.0287686)
Supplement: S4 Table — (DOCX) [file pone.0287686.s005.docx]

| S4 Table. Results of mediation analysis assessing if perinatal distress is a mediator in the pathway between socioeconomic adversity and adverse birth outcome | | | | | | | |
| --- | --- | --- | --- | --- | --- | --- | --- |
| For LBW as adverse birth outcome and total perinatal stress score as a mediator | **Average direct effect** | ***P*-value** | **Average causal mediated effect** | ***P*-value** | **Total effect** | ***P*-value** | **Proportion mediated** |
|  | **Coefficient (95% CI)** |  | **Coefficient (95% CI)** |  | **Coefficient (95% CI)** |  |  |
| Wealth index |  |  |  |  |  |  |  |
| Lowest | -0.008 (-0.079, 0.090) | .794 | 0.013 (0.004, 0.030) | **.002** | -0.006 (-0.063, 0.100) | .946 | 15.6% |
| Low | -0.044 (-0.112, 0.030) | .254 | 0.009 (0.001, 0.020) | **.016** | -0.033 (-0.098, 0.050) | .368 | 12.9% |
| Middle | -0.011 (-0.080, 0.070) | .760 | 0.006 (-0.001, 0.020) | .110 | -0.004 (-0.071, 0.070) | .890 | 2.1% |
| High | 0.010 (-0.063, 0.100) | .820 | 0.007 (-0.002, 0.020) | .120 | 0.016 (-0.054, 0.100) | .690 | 9.8% |
| Highest | Reference | **-** | Reference | **-** | Reference | **-** |  |
| Not empowered women, yes | 0.078 (0.004, 0.130) | **.040** | 0.002 (-0.007, 0.010) | .696 | 0.079 (0.003, 0.130) | **.038** | 1.8% |
| Food insecurity, yes | 0.023 (-0.035, 0.080) | .460 | 0.023 (0.011, 0.040) | **.000** | 0.043 (-0.008, 0.100) | .096 | 49.4% |
| Intimate partner violence, yes | 0.082 (0.013, 0.160) | **.024** | 0.016 (0.005, 0.030) | **.002** | 0.092 (0.025, 0.170) | **.006** | 17.1% |
| Low social support, yes | 0.157 (0.037, 0.310) | **.012** | 0.050 (0.013, 0.090) | **.004** | 0.184 (0.074, 0.330) | **.002** | 27.9% |
| At least one stressful life event, yes | 0.002 (-0.048, 0.050) | .954 | 0.005 (-0.0001, 0.010) | .064 | 0.007 (-0.042, 0.060) | .808 | 9.7% |
| For SGA as adverse birth outcome and total perinatal stress score as a mediator | **Average direct effect** | ***P*-value** | **Average causal mediated effect** | ***P*-value** | **Total effect** | ***P*-value** | **Proportion mediated** |
|  | **Coefficient (95% CI)** |  | **Coefficient (95% CI)** |  | **Coefficient (95% CI)** |  |  |
| Wealth index |  |  |  |  |  |  |  |
| Lowest | -0.035 (-0.110, 0.050) | .420 | 0.013 (0.004, 0.030) | **.002** | -0.019 (-0.092, 0.070) | .608 | 18.2% |
| Low | -0.007 (-0.089, 0.090) | .786 | 0.012 (0.003, 0.030) | **.012** | 0.005 (-0.073, 0.100) | .958 | 8.9% |
| Middle | 0.015 (-0.064, 0.110) | .780 | 0.008 (-0.001, 0.020) | .090 | 0.023 (-0.056, 0.120) | .630 | 12.6% |
| High | 0.019 (-0.063, 0.110) | .700 | 0.008 (-0.002, 0.020) | .120 | 0.026 (-0.056, 0.120) | .590 | 11.1% |
| Highest | Reference | **-** | Reference | **-** | Reference | **-** |  |
| Not empowered women, yes | 0.081 (0.002, 0.150) | **.048** | 0.002 (-0.007, 0.010) | .718 | 0.082 (0.004, 0.150) | **.042** | 1.9% |
| Food insecurity, yes | 0.051 (-0.011, 0.110) | .100 | 0.025 (0.011, 0.040) | **.000** | 0.071 (0.016, 0.130) | **.010** | 7.1% |
| Intimate partner violence, yes | 0.135 (0.063, 0.220) | **.000** | 0.019 (0.006, 0.040) | **.000** | 0.146 (0.073, 0.230) | **.000** | 12.6% |
| Low social support, yes | 0.193 (0.059, 0.350) | **.008** | 0.054 (0.014, 0.100) | **.010** | 0.223 (0.097, 0.370) | **.000** | 25.1% |
| At least one stressful life event, yes | -0.008 (-0.063, 0.050) | .770 | 0.013 (0.005, 0.020) | **.000** | 0.005 (-0.048, 0.060) | .890 | 25.2% |
