# Supplementary material for: Influence of perinatal distress on adverse birth outcomes: A prospective study in the Tigray region, northern Ethiopia
Source: PLoS One. 2023 Jul 13;18(7):e0287686. doi: 10.1371/journal.pone.0287686 (PMC10343148; doi:10.1371/journal.pone.0287686)
Supplement: S2 Checklist — (DOCX) [file pone.0287686.s008.docx]

STROBE Statement—checklist of items that should be included in reports of observational studies

|  | Item No. | Recommendation | Page No. | Relevant text |
| --- | --- | --- | --- | --- |
| **Title and abstract** | 1 | (*a*) Indicate the study’s design with a commonly used term in the title or the abstract | Page 1 line 2, and Page 2 lines 35-37 |  |
|  |  | (*b*) Provide in the abstract an informative and balanced summary of what was done and what was found | Page 2 lines 35-48 |  |
| Introduction | | | |  |
| Background/rationale | 2 | Explain the scientific background and rationale for the investigation being reported | Page 4 lines 65-86, and Page 4 lines 87-91, and 96-104 |  |
| Objectives | 3 | State specific objectives, including any prespecified hypotheses | Page 5 lines 105-107 |  |
| Methods | | | |  |
| Study design | 4 | Present key elements of study design early in the paper | Page 5 lines 110-111, and Page 6 lines 9-11 |  |
| Setting | 5 | Describe the setting, locations, and relevant dates, including periods of recruitment, exposure, follow-up, and data collection | Page 6 lines 17-19, and Page 5 lines 14-15 |  |
| Participants | 6 | (*a*) *Cohort study*—Give the eligibility criteria, and the sources and methods of selection of participants. Describe methods of follow-up  *Case-control study*—Give the eligibility criteria, and the sources and methods of case ascertainment and control selection. Give the rationale for the choice of cases and controls  *Cross-sectional study*—Give the eligibility criteria, and the sources and methods of selection of participants | Page 6 lines 137-138  Not applicable  Not applicable |  |
|  |  | (*b*) *Cohort study*—For matched studies, give matching criteria and number of exposed and unexposed  *Case-control study*—For matched studies, give matching criteria and the number of controls per case | Not applicable  Not applicable |  |
| Variables | 7 | Clearly define all outcomes, exposures, predictors, potential confounders, and effect modifiers. Give diagnostic criteria, if applicable | Page 7 lines 147-164, Page 8 lines 165-192, Page 9 193-219, page 10 lines 221-248, and Page 11 lines 249-250 |  |
| Data sources/ measurement | 8* | For each variable of interest, give sources of data and details of methods of assessment (measurement). Describe comparability of assessment methods if there is more than one group | Page 7 lines 147-164, Page 8 lines 165-192, Page 9 lines 193-219, Page 10 lines 221-248, and Page 11 lines 249-250 |  |
| Bias | 9 | Describe any efforts to address potential sources of bias | Page 12 lines 281-286 |  |
| Study size | 10 | Explain how the study size was arrived at | Page 6 lines 123-130 |  |

| Quantitative variables | | 11 | Explain how quantitative variables were handled in the analyses. If applicable, describe which groupings were chosen and why | Page 12 lines 279-280 | |  |
| --- | --- | --- | --- | --- | --- | --- |
| Statistical methods | | 12 | (*a*) Describe all statistical methods, including those used to control for confounding | Page 11 lines 252-277, and Page 12 lines 278-280 | |  |
|  |  |  | (*b*) Describe any methods used to examine subgroups and interactions | Page 11 line 277, and Page 12 lines 278-279 | |  |
|  |  |  | (*c*) Explain how missing data were addressed |  | |  |
|  |  |  | (*d*) *Cohort study*—If applicable, explain how loss to follow-up was addressed  *Case-control study*—If applicable, explain how matching of cases and controls was addressed  *Cross-sectional study*—If applicable, describe analytical methods taking account of sampling strategy | Page 12 lines 295-297 | |  |
|  |  |  | (*e*) Describe any sensitivity analyses |  | |  |
| Results | | | | | | |
| Participants | | 13* | (a) Report numbers of individuals at each stage of study—eg numbers potentially eligible, examined for eligibility, confirmed eligible, included in the study, completing follow-up, and analysed | S1 Fig | |  |
|  |  |  | (b) Give reasons for non-participation at each stage | S1 Fig | |  |
|  |  |  | (c) Consider use of a flow diagram | S1 Fig | |  |
| Descriptive data | | 14* | (a) Give characteristics of study participants (eg demographic, clinical, social) and information on exposures and potential confounders | Tables 1 and 2 | |  |
|  |  |  | (b) Indicate number of participants with missing data for each variable of interest | S1 Fig | |  |
|  |  |  | (c) *Cohort study*—Summarise follow-up time (eg, average and total amount) | Page 12 line 294 | |  |
| Outcome data | | 15* | *Cohort study*—Report numbers of outcome events or summary measures over time | Table 1(see row 1) | |  |
|  |  |  | *Case-control study—*Report numbers in each exposure category, or summary measures of exposure | Not applicable | |  |
|  |  |  | *Cross-sectional study—*Report numbers of outcome events or summary measures | Not applicable | |  |
| Main results | | 16 | (*a*) Give unadjusted estimates and, if applicable, confounder-adjusted estimates and their precision (eg, 95% confidence interval). Make clear which confounders were adjusted for and why they were included | Page 18 Table 3 and , and lines 339-341 | |  |
|  |  |  | (*b*) Report category boundaries when continuous variables were categorized | Page 7 lines 147-164, Page 8 lines 165-192, Page 9 lines 193-219, Page 10 lines 221-248, and Page 11 lines 249-250 | |  |
|  |  |  | (*c*) If relevant, consider translating estimates of relative risk into absolute risk for a meaningful time period | Not relevant | |  |
| Other analyses | 17 | | Report other analyses done—eg analyses of subgroups and interactions, and sensitivity analyses | Page 19 lines 346-349 |  | |
| Discussion | | | | | | |
| Key results | 18 | | Summarise key results with reference to study objectives | Page 21 lines 354-363 |  | |
| Limitations | 19 | | Discuss limitations of the study, taking into account sources of potential bias or imprecision. Discuss both direction and magnitude of any potential bias | Page 22 lines 399-407 |  | |
| Interpretation | 20 | | Give a cautious overall interpretation of results considering objectives, limitations, multiplicity of analyses, results from similar studies, and other relevant evidence | We think highlighting the limitations is adequate |  | |
| Generalisability | 21 | | Discuss the generalisability (external validity) of the study results | Page 21 lines 354-363 |  | |
| Other information | | |  | | | |
| Funding | 22 | | Give the source of funding and the role of the funders for the present study and, if applicable, for the original study on which the present article is based | Page 23 line 417 |  | |

*Give information separately for cases and controls in case-control studies and, if applicable, for exposed and unexposed groups in cohort and cross-sectional studies.

**Note:** An Explanation and Elaboration article discusses each checklist item and gives methodological background and published examples of transparent reporting. The STROBE checklist is best used in conjunction with this article (freely available on the Web sites of PLoS Medicine at http://www.plosmedicine.org/, Annals of Internal Medicine at http://www.annals.org/, and Epidemiology at http://www.epidem.com/). Information on the STROBE Initiative is available at www.strobe-statement.org.
